# Incidence of Renal Involvement in Malaria in Children of Odisha

**DOI:** 10.5402/2013/573735

**Published:** 2012-11-01

**Authors:** Rajesh Kumar Padhi, Soumya Mishra

**Affiliations:** ^1^Department of Pediatrics, Kalinga Institute of Medical Sciences, KIIT Campus-5, Patia, Odisha, Bhubaneswar 751024, India; ^2^Department of Physiology, Kalinga Institute of Medical Sciences, KIIT Campus-5, Patia, Odisha, Bhubaneswar 751024, India

## Abstract

About 500 million people suffer from malaria leading to death in 2-3 million cases every year, of which about 1 million are children. Horstman et al., 1985, and Weber et al., 1991, demonstrated an acute renal failure as a well-described complication of *Plasmodium falciparum* malaria in nonimmune adults and a major contributor to their mortality. In children, renal failure, though not very common, has become a rising issue leading to death. This study aims at determining the incidence of renal complication in malaria cases reported in children of Odisha. 108 cases of malaria who were admitted to Department of Paediatrics, SCB Medical College and Hospital and Sardar Vallab Bhai Patel Post Graduate Institute of Paediatrics, Cuttack, Odisha, India during the period from July 2006 to November 2008 were included in the prospective study. Extensive investigations were carried out to check for renal involvement in these cases. 50.9% of cases showed some form of renal involvement, most of which were recorded in age group of 5–10 years. Overall, males had a higher incidence than females. 62.7% of total cases infected with *P. falciparum* showed renal involvement though mixed infections with both *P. falciparum* and *P. vivax* had 100% renal involvement.

## 1. Introduction

Malaria is now a leading public health problem in developing world including India. Almost all complications and death from malaria are caused by *Plasmodium falciparum* [1]. Recently there is a changing trend not only in the clinical manifestation but also in the pattern of complications due to malaria. Over a decade ago, cerebral malaria was the predominant manifestation of severe malaria, where as today the combination of jaundice and renal failure is more common [2]. Some studies demonstrated acute renal failure as a well described complication of P.falciparum malaria in non-immune adults and a major contributor to their mortality [3] demonstrated acute renal failure as a well-described complication of *P. falciparum* malaria in non-immune adults and a major contributor to their mortality. In contrast major manifestations of severe *falciparum* malaria in children are cerebral malaria, severe anemia, metabolic acidosis [4], but renal failure is not commonly encountered [5].

The clinical spectrum of renal involvement in malaria varies widely from urinary sediment abnormalities, mild proteinuria, electrolyte changes to acute renal failure (ARF) with metabolic acidosis as well as nephritic syndrome. Among renal manifestations, ARF is the commonest manifestation of severe *falciparum* malaria. The overall incidence of ARF reported by various Indian authors ranges between 4 and 17.2% [6]. Studies have shown that incidence of ARF (serum creatinine > 3 mg%) in pediatric population with complicated *falciparum* malaria is 7.7% in under-5 children and 18.4% in 5–14-year children [7]. 

The clinical syndrome of quartan malarial nephropathy [8] is nonspecific, apart from its association with the features of the parasitic infection. The majority of patients are children with a mean age of 5 years. Proteinuria is encountered in a variable proportion of patients, up to 46% in the first published series. Microhematuria is occasionally noted, particularly in the older age groups. Overt nephritic syndrome develops in a yet-undefined fraction, and hypertension is a late symptom. The disease is progressive despite successful eradication of the infection culminating in chronic renal failure over 3–5 years.

*P. falciparum* nephropathy can manifest as acute tubular necrosis, acute interstitial nephritis, and glomerulonephritis. The incidence of acute tubular necrosis is estimated at between 1 and 4% of all cases of *falciparum* malaria, but it may reach 60% in cases clinically categorized as being malignant. Prognosis depends on the severity of the condition, associated extrarenal complication, response to anti-parasitic treatment and availability of dialysis. The reported mortality ranges from 15 to 30% [9–11].

Although isolated interstitial nephritis has not been reported in humans, interstitial inflammation is a common histopathological finding in *P. falciparum* acute tubular necrosis and acute glomerular nephritis [12, 13]. Glomerular injury may be associated with *falciparum* malaria at any age, although children remain the main population at risk [14–16]. It is impossible to estimate the true incidence since the disease is essentially mild, transient and overshadowed, by the other complications. Nephrotic [17] and acute nephritic [18, 19] syndromes are occasionally seen. In contrast to quartan malarial nephropathy, *falciparum* glomerulopathy is reversible within 2–6 weeks upon the eradication of the infection.

Resurgence of malaria in Odisha provided an opportunity to study the different renal manifestation of malaria in children and its correlation with severity of infection and clinical presentation. This study aims at determining the incidence and renal involvement in different types of malaria.

## 2. Materials and Methods

The study was conducted in the Department of Paediatrics, SCB Medical College and Hospital and Sardar Vallab Bhai Patel Post Graduate Institute of Paediatrics, Cuttack, Odisha, India from July 2006 to November 2008. All cases admitted to the paediatric ward with smear-positive or ICT-positive malaria were taken up as subjects for the prospective study. Patients with preexisting renal disease were excluded from the study. Cases with clinical features of malaria with asexual form of plasmodium in the peripheral smear seen by thick and thin smear were taken into consideration. It included 108 cases of malaria, diagnosed either by smear examination or rapid diagnostic test. Other investigations like hematological tests, biochemical tests, and urine examination were done. Smearing was done under aseptic conditions and stained to examine under oil immersion objective of light microscope. The nucleus of the malaria parasite looks pink and cytoplasm blue in color. Thick blood film increases sensitivity of the parasite detection because of more parasite concentration even though the morphology of parasite is distorted. One microscope field is equivalent to 50 microscope fields of thin film. Hence it is more useful for mass surveys and a guide to assess the antimalarial response.

*P. falciparum* was identified by the following characteristic features:

- two nuclei on same side of the ring/opposite side of ring,
- multiple infection of RBC,
- accole form of trophozoite,
- the size of the infected RBC does not alter,
- sickle-shaped gametocytes which are one and half size of RBC.

*P. vivax* was identified by the following characteristic features:

- one parasite in a RBC,
- cytoplasm is thick with single nucleus,
- parasitized RBC is larger than normal,
- accole is not seen.

Detailed evaluation was done with special reference to urine output and presence of edema. All cases were subjected to investigations such as serum urea, creatinine, electrolyte, and urine analysis to check for renal involvement. Deviations from the normal range of variables are extrapolated as markers of renal pathology. Blood urea nitrogen and creatinine ratio was calculated for each patient from the tabulated data and it was inferred whether the renal involvement was due to prerenal causes or of renal etiology. If urea creatinine ratio was >20 mg/dL, then it was considered as pre-renal cause and urea creatinine <20 mg/dL as renal causes.

Table 1 shows the criteria considered for classifying as renal impairment.

If any one of these above criteria is fulfilled, then it is considered as renal involvement present.

## 3. Results

**Figure 1:**
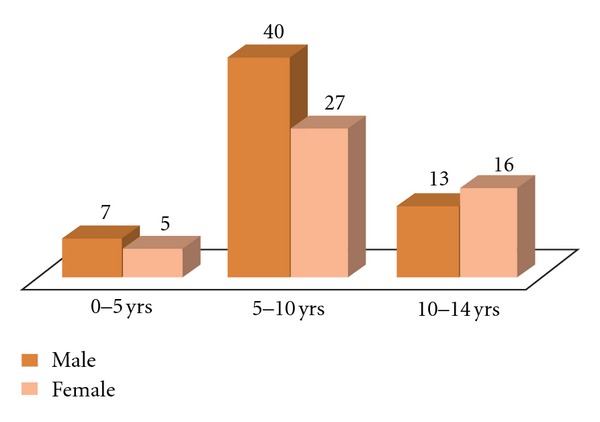
Age and sex distribution of malaria cases seen.

Out of the 108 cases studied, there were 60 (55.5%) male and 48 (44.5%) female cases with a male-to-female ratio of 1.25 : 1. This coincides with the fact that male children are usually less covered and exposed more to mosquito bites. A maximum number of cases were detected in the age group of 5–10 years (62%) followed by 10–14 years (27%), and 0–5 years (11%).  Figure 1 shows age and sex distribution of the total cases.

**Figure 2:**
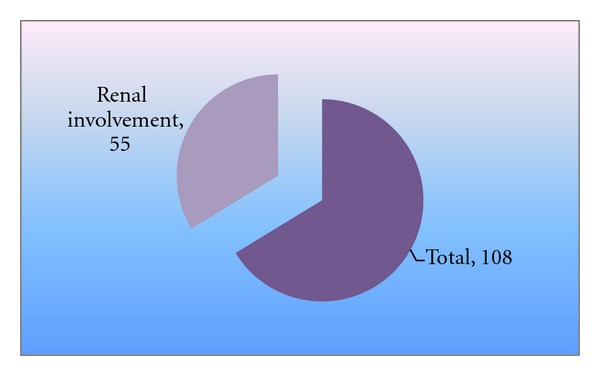
Renal involvement out of total cases.

As seen from Figure 2, there were 55 cases (50.9%) which have renal involvement in the form of proteinuria, increased serum creatinine, abnormal urinary casts, cells, and so forth. Maximum incidence of renal involvement was in the 5–10 years age group. Repeated malaria infection causing immunological injury to the kidney may be the possible explanation. 

**Figure 3:**
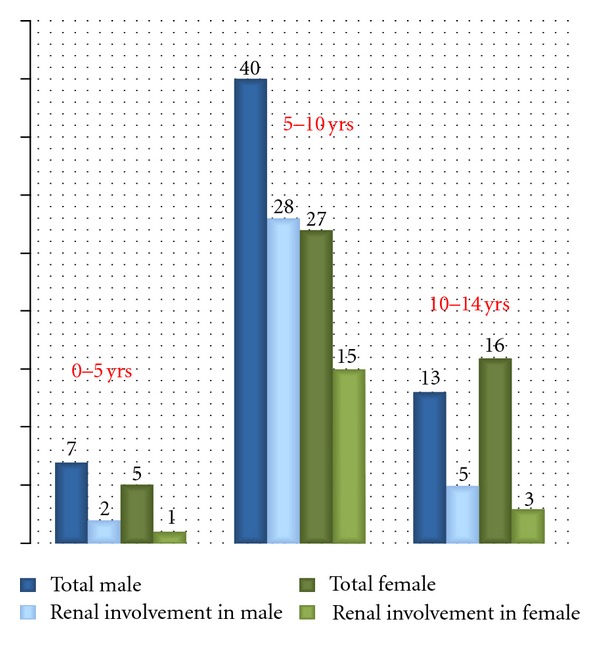
color difference of females—total females and females with renal involvement is not very distinguishable.

Figure 3 depicts the age and sex distribution of renal involvement. Most of the renal involvement is seen in the age group of 5–10 years in both males and females though male children are more affected in all age groups than the female children.

**Figure 4:**
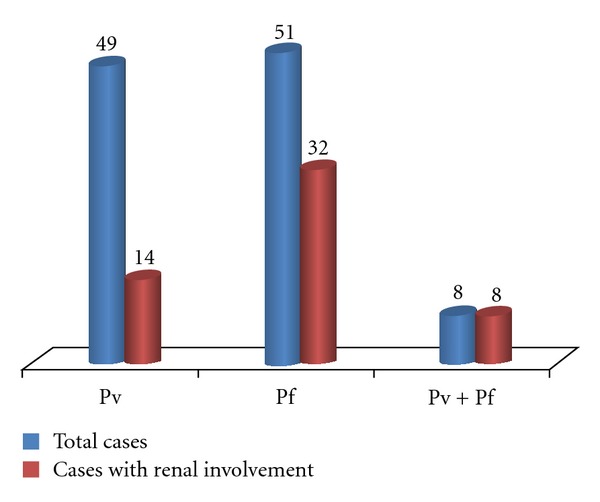
Renal involvement according to type of malaria.

62.7% of patient with *falciparum* malaria has renal involvement as compared to only 28.6% of vivax cases. All mixed infection cases have renal involvement.  Figure 4 shows the type of malaria and its relation with increased urinary protein excretion.

## 4. Discussion

Malaria reemerged once again as a major public health problem of India in the late 1970s after a significant decline in the 60's, affecting 2–2.5 million cases usually. Thus malaria is a parasitic diseases of great epidemiological importance, and malarial renal involvement is emerging as an important problem in tropical countries. Renal involvement in malaria displays the full spectrum of interaction between red-cell abnormalities and TH1 and TH2 activation. At one extreme is a slowly progressive immune-complex mediated glomerulopathy that supervenes in a setting of TH2 predominance, classically associated with *P. malaria* infection. At the other extreme is a severe disease dominated by the hemodynamic consequences of massive red-cell parasitization, eventually leading to acute tubular necrosis. This is mostly seen with *P. falciparum* infection. In between lay two syndromes, namely, acute interstitial nephritis and acute proliferative glomerulonephritis.

In the study 50.9% have some form of renal involvement. Similar results were seen in different studies, 30–70% [20], 46.9% [21], 53–46% [22]. In different studies, the male to female ratio varies from 2 : 1 [23] to 1.6 : 1 [24]. The sex difference in incidence of malaria may be because of different medical-care-seeking behavior in different socioeconomic status, ethnic groups, and attitude of parents especially mothers towards male children. Also more outdoor activity of male children may be a contributory factor.

In another study, maximum cases observed in age group 0–5 years [25]. But in the present study maximum number of cases is seen in the age group of 5–10years. From late infancy to two year the child has no immunity and is no longer protected by mother's antibodies. Thereafter even though immunity develops it is weak till age of 5 years. This explains why the maximum incidence of malaria in this study observed is in the said age group [26].

 In this study 62.7% of patient cases have some form of renal involvement. Similarly other studies have given comparable results. Few studies give 58.2% of renal involvement in falciparum malaria, 23% in vivax malaria [22]. The percentage of renal involvement is maximum in mixed infection group. The hemodynamic changes are more malignant in case of falciparum malaria as the RBC parasitization rate and micro-vascular obstruction is maximum in falciparum malaria [3].

## 5. Conclusion

It is concluded that male children of 5–10 years are more vulnerable for malaria with renal involvement more so if mixed infection is present. Early and prompt diagnosis along with aggressive therapy can prevent progression to complicated malaria while we are waiting to combat other factors like vector control, administrative shortfalls, financial stringency, and so forth. A new molecular marker has to be searched for, and that may give early clue to renal involvement before biochemical abnormality of renal involvement is detected.

## Figures and Tables

**Table 1:** Criteria for classifying as renal impairment.

Creatinine	>1.5 mg/dL or rise of >0.3 mg/dL from baseline level (if available)
Hematuria	>5 RBC/high power field
Proteinuria	>traces by dip stick method
Cast	Granular cast, RBC cast, and muddy brown cast
Edema	Periorbital edema, pitting edema of lower extremities
Anuria	No urination
Oliguria	<0.5 mL/kg/hr urination (at least for 6 hours)
